# The Year's Work as Shown in Local Reports

**Published:** 1910-07-16

**Authors:** 


### Public Health and Hygiene.

### THE YEAR'S WORK AS SHOWN IN LOCAL REPORTS.

Borough of Royal Leamington Spa. Second annual report of the School Medical Officer (to December 31, 1909). (Leamington Spa: A. Tomes. 1910. Pp. 55.)

From Dr. Burnet's report we gather that 3,509 children have been examined during the year. Of these, decayed teeth (four or more) were found in 22.1 per cent., enlarged tonsils in 18.1 per cent., adenoids in 8 per cent., dirty heads in 5.4 per cent. of boys and 9.3 per cent. of girls, dirty bodies in 8.8 per cent. of boys and 6.9 per cent. of girls, defective eyesight in 7.7 per cent. of both sexes, there being only three more cases found in girls than in boys. A curious feature is the prevalence of enlarged thyroids at one of the schools, no less than 21.7 per cent. of the children being affected. At the other schools the average is 2 per cent. This disparity cannot be accounted for by the hardness of the water, for the supply in the most heavily affected district is softer and contains less solids than that of other districts. Neither can it be explained on the ground of heredity or an excessive consumption of flesh-meat. The author has been attempting to find out the influence of complexion on disease. He has been unable to substantiate the statement often made that during childhood disease falls more heavily on the fair element. His results lead him to find that the dark element are the sufferers.

Annual Report of the School Medical Officer for the Year 1909. The Devon County Education Committee. (Exeter: Wm. Pollard and Co., Ltd. 1910. Pp. 60.)

In this interesting report Dr. George Adkins, County Medical Officer of Health and School Medical Officer, gives an account of the first year's working of the medical inspection of school children. During the year all schools were examined by the medical inspection staff, except those closed for infectious disease. In the county there are 544 schools with 652 departments and an average attendance of 57,383 children. Of these last, 21,063 were examined and 12,642 found defective. The ages of the children examined varied from three to seven years for entrants, and twelve to fourteen years for leavers. Of defective children the largest proportion was represented by those suffering from pediculosis, the percentage of cases reported working out at about 3.9 for boys and 40.8 for girls. The next largest group consisted of those suffering from defective vision, the percentages being about 13.4 for boys and 17.2 for girls. Defective teeth accounted for 15.6 per cent. in boys and 13.8 per cent. in girls. Of previous illnesses measles was the most prevalent, next to which came whooping cough, mumps ranking third on the list. Statistics from which these results are compiled are not, however, reliable, as it was difficult to get accurate information from parents. It has been found possible to examine ten children in an hour; but only one ordinary visit has been made to each school in the year, as the present staff of three full-time and one part-time officers is not sufficient to allow of more frequent visits being paid except for cases specially reported. It is difficult to arrive at the exact cost of this medical inspection; but the salaries of medical officers amounted to £803, with travelling expenses £200 17s., and the cost of measuring instruments, surgical appliances, &c., was £168 12s. 5d. The school medical officer receives no salary as such. Dr. Adkins states that on the whole the medical inspection has worked smoothly, very little friction having occurred with parents, 31.6 per cent. of whom were present at, and only 5 per cent. of children (either through illness or objection to the examination) absent from, the inspections.

Annual Report of the Medical Officer of Health of the County Borough of Warrington on the Sanitary Condition of Warrington, with Tabular Returns of the Sickness and Mortality during the Year 1909; also the Report of the Inspector of Nuisances. (Warrington: Mackie and Co., Ltd. 1910. Pp. 62).

We have gathered from Dr. Coote-Hibbert's report that the general, zymotic, and infantile death-rates in the borough for 1909 were respectively 18.2, 2.9, and 129 per thousand, as compared with average rates of 15.6, 1.42, and 118 per thousand in the seventy-six large towns. The borough rates, while not comparing favourably with those of the other large towns, do not however show much difference from those of the previous year. In 1908 the high zymotic rate was due to an epidemic of whooping-cough which accounted for 60 deaths, whereas in 1909 an epidemic of measles caused 102 deaths and sensibly raised the number of deaths from pneumonia, which rose from 104 in 1908 to 140 in 1909. Scarlet fever, diphtheria, and enteric fever were respectively responsible for 21, 26, and 15 deaths, as compared with 16, 16, and 11 deaths in 1908. The Public Health (Tuberculosis) Regulations 1908, which came into force on January 1, 1909, resulted in 135 cases of phthisis being notified. The medical officer thinks that a rough estimate from averages of the real number of cases of phthisis in the borough would be about 370. The marriage-rate per thousand worked out at 8.4, as against 8.8 for the year before. The birth-rate was 30.9, as against 25.6, the general rate for England and Wales. The water-supply is good, and bacteriological examination has shown that it is extremely pure. Dr. Hibbert utters a word of warning as to the increasing number of children annually exempted from vaccination, the cases of exemption having risen from three in 1905 to 148 in 1910. A number of well-printed statistical charts, many of which are in colours, add to the value of the report.

City of Coventry: Annual Report on the Health of the City. By E. H. Snell, M.D., B.Sc. Lond., F.R.S. Edin., Barrister-at-Law, Medical Officer of Health. (Coventry: Curtis and Beamish, Ltd. 1910. Pp. 202).

The city of Coventry had an estimated population of 93,500 for the year 1909. Taking this estimate as the basis upon which to form the calculations, the general, infantile, and zymotic death-rates per 1,000 of population worked out respectively at 13.7, 96.8, and 1.6, with a mean age at death of 35.1 years. The corresponding average rates for the previous ten years were respectively 15.2, 12.4, and 1.7. The marriage-rate was 17, and the birth-rate 27.8, the respective figures for the previous decade being 16.8 and 28.9. No cases of smallpox were notified during the year, but 1,070 certificates of exemption from vaccination were granted. During the same period 2,601 births were notified and 1,385 babies vaccinated. Thus only 53.2 per cent. of infants were submitted to vaccination, a fact which the medical officer cannot help remarking upon, despite the unsuitability of a report as a place in which to discuss such a controversial subject. He ventures to remark that in the event of an epidemic of smallpox the percentage of vaccinations would certainly increase, tending to show that the objections are not entirely of a conscientious character. During the year 121 cases of diphtheria were notified, from which disease 11 died. Phthisis accounted for 97 deaths, and other forms of tuberculosis for 37, giving a tuberculosis death-rate of 1.43. Deaths resulting from other diseases were : Cancer 65, whooping-cough 29, erysipelas 3, puerperal fever 1, enteric fever 4, scarlet fever 24, measles 67, epidemic diarrhœa 18, alcoholism 1, cirrhosis of the liver 21, cerebro-spinal fever 2, accident or negligence (including homicide and suicide) 49, cause ill-defined 1, and uncertified 33. There were buried in the Coventry cemeteries 113 bodies of infants said to have been stillborn, of which 58 occurred in the practice of medical men and 55 in that of midwives. The rainfall for the year was 26.65 inches, and the number of rain-days 218. Under the Midwives Act medical aid was summoned in 227 cases, out of 1,964 attended by midwives. The second portion of the report deals with the medical inspection of schools and school children. The total number of children examined was 4,838. Out of 1,560 children inspected to ascertain the condition of vision, 17.3 per cent. were found to have defective eyesight, 14.8 in boys and 19.5 in girls. Only 13 per cent. of children had mouths obviously free from carious teeth. Pediculi capitis were found in 7.8 per cent. of boys and 45.4 per cent. of girls. In 25.6 per cent. of both sexes enlarged tonsils were noticed, and external ear diseases in 1.5 per cent. Other diseases were seen in the following percentages. Rickets 5, skin diseases 3.2, heart disease 1, lung disease 7, tuberculosis 0.51, and deformities of various kinds 7.8. In Part III. a report is given of the general sanitary administration, including isolation hospitals, housing, town-planning, slaughter-houses, lodging-houses (both private and common), offensive trades, and the sale of foods and drugs. The volume ends with a report on the utility of hospital accommodation for diphtheria, and that of the deputation who attended the Leeds Health Congress.

County Borough of Bournemouth. Annual Report of the Medical Officer of Health, 1909, with which are included the reports of the Borough Bacteriologist, Chief Sanitary Inspector, and Public Analyst. (Bournemouth : The Richmond Hill Printing Works, Ltd. 1910. Pp. 71.)

Standing on a sandy subsoil, with an area of 5,850 acres, the County Borough of Bournemouth has an estimated population of 79,288. Its general, infantile, and zymotic death-rates for 1909 stood respectively at 11.46, 91.43, and 0.21 per thousand. The birth-rate was 11.46 per 1,000. The following diseases were responsible for the number of deaths added after them : Measles, 7; scarlet fever, 3; whooping-cough, 5; diphtheria, 9; influenza, 2; diarrhœa, 10; enteritis, 6; gastritis, 5; puerperal fever, 1; erysipelas, 4; phthisis, 150; tubercle elsewhere, 12; cancer, 75; bronchitis, 70; pneumonia, 47; alcoholism, 4; venereal disease, 3; parturition, 5; heart disease, 178; accidents, 14; suicides, 4; prematurity, 22; all other causes, 273. The water-supply of the borough has been found quite satisfactory by both chemical and bacteriological examinations.

Annual Report of the Statistics and Sanitary Condition Relating to the City of Westminster for the Year 1909. By Francis J. Allan, M.D., C.M., D.P.H. Cantab., F.R.S.Edin., Medical Officer of Health. (London : Harrison and Sons. 1910. Pp. 118.)

The City of Westminster has an estimated resident population of 172,150. Its general, infantile, and zymotic death-rates during the year were, respectively, 12.7, 93, and 0.31 per thousand. The report contains elaborate details as to the causes of death, which were as follows : Smallpox, 1; measles, 26; scarlet fever, 18; influenza, 43; whooping cough, 23; diphtheria, 18; cerebro-spinal meningitis, 1; enteric fever, 9; diarrhœa, 6; enteritis, 23; erysipelas, 6; puerperal fever, 2; septic diseases, 17; syphilis, etc., 15; diabetes, 23; gout, 8; rheumatic fever, 7; tubercle, 53; phthisis, 225; alcohol, 23; cirrhosis of liver, 38; cancer, 192; premature birth, 64; developmental diseases, 64; meningitis, 12; nervous diseases, 95; old age, 103; heart disease, 87; pneumonia, 121; respiratory disease, 66; digestive disorders, 54; Bright's disease, 117; generative system disease, 5; parturition, 5; accident, 62; suicide and murder, 25; and other causes, 35. The birth-rate was 15.9 per thousand, 2,751 children being born, of whom 203 were illegitimate. In Part II. of the report, which deals mainly with sanitation and the laws and by-laws relating thereto, mention is made of an epidemic of scarlet fever which occurred on June 16 and 17, and which was traced to infectious milk. This milk was found to have been obtained from one farm. On the day when it developed its infectious qualities, the milk had had added to it that of three cows which had recently calved, the calf of one of which had died after being suckled for four or five days. The three cows were all in a morbid condition, a fact which renders it probable that a morbid condition of the cows was responsible for the human illness which ensued in the present case. In all, forty-three people were taken ill as the result of drinking this milk. Later in the report there is an interesting account of Dr. Bernstein's experiments with boric acid as a food preservative, undertaken at the instigation of the medical officer of health, who was defending an appeal against a conviction in 1908 under the Sale of Food and Drugs Act.

Fifteenth Report of the Board of Health of the Town of Montclair, New Jersey, from January 1 to December 31, 1909. By Chester H. Wells, Health Officer and Registrar of Vital Statistics. (Publisher not mentioned. Pp. 69.)

It is interesting, while on the subject of health reports, to compare figures derived from statistics of an American town with those of our own country's cities and towns. The town of Montclair, New Jersey, has a population of 18,297, of whom 2,195 are coloured people and 1,650 Italians. The corrected death-rate for the town in 1909 was 11.48, the infantile and zymotic death-rates being, respectively, 82 and 0.44 per thousand. The chief causes of death were as follows : Pneumonia, 21; cardiac disease, 19; cerebral hæmorrhage, 18; phthisis, 15; Bright's disease, 15; cancer, 12; acidents and suicides, 11; diarrhœa, 10; infantile disorders, 10; infectious diseases, 8; and appendicitis, 6. The corrected birth-rate worked out at 23.2. The Health Officer draws attention to the fact that the last report of the State Board of Health contains the statement that in a recent investigation of the ages of 4,000 American-born children it was found that public records of the birth were available for less than two-fifths of the children. He points out that negligence in the certification of births often leads to legal trouble in after-life when the question of inheritance, of child labour, of the requirements of foreign countries, of the disabilities of miners, etc., may crop up. Children to the number of 3,793 were examined in the schools, of whom 4.5 per cent. suffered from eye disease, 8.3 from nose and throat affections, only 14 per cent. being passed as healthy. The Health Officer also calls attention to the lack of sanatoria for the treatment of early cases of phthisis, there being but one hospital of 120 beds to supply the whole State. The marriage-rate for the town was 8.2, there being only one case of a white man marrying a coloured woman. The efficient way in which the milk-supply is dealt with is made manifest by the perusal of the elaborate tables of the samples taken from each dealer at least once a month, with their analysis, which bring the volume to a close.

